# Functional connectivity abnormalities of the long-axis hippocampal subregions in schizophrenia during episodic memory

**DOI:** 10.1038/s41537-021-00147-2

**Published:** 2021-03-03

**Authors:** Jules R. Dugré, Alexandre Dumais, Andras Tikasz, Adriana Mendrek, Stéphane Potvin

**Affiliations:** 1 Centre de Recherche de l’Institut Universitaire en Santé Mentale de Montréal, Montreal, QC Canada; 2 Department of Psychiatry and Addiction, Faculty of Medicine, University of Montreal, Montreal, QC Canada; 3 Institut National de Psychiatrie Légale Philippe-Pinel, Montreal, QC Canada; 4 Department of Psychology, Bishop’s University, Sherbrooke, QC Canada

**Keywords:** Schizophrenia, Schizophrenia

## Abstract

Past evidence suggests that hippocampal subregions, namely the anterior and posterior parts, may be engaged in distinct networks underlying the memory functions which may be altered in patients with schizophrenia. However, of the very few studies that have investigated the hippocampal longitudinal axis subdivisions functional connectivity in patients with schizophrenia, the majority was based on resting-state data, and yet, none aimed to examine these during an episodic memory task. A total of 41 patients with schizophrenia and 45 healthy controls were recruited for a magnetic resonance imaging protocol in which they performed an explicit memory task. Seed-based functional connectivity analysis was employed to assess connectivity abnormalities between hippocampal subregions and voxel-wise connectivity targets in patients with schizophrenia. We observed a significantly reduced connectivity between the posterior hippocampus and regions from the default mode network, but increased connectivity with the primary visual cortex, in patients with schizophrenia compared to healthy subjects. Increased connectivity between the anterior hippocampus and anterior temporal regions also characterized patients with schizophrenia. In the current study, we provided evidence and support for studying hippocampal subdivisions along the longitudinal axis in schizophrenia. Our results suggest that the abnormalities in hippocampal subregions functional connectivity reflect deficits in episodic memory that may be implicated in the pathophysiology of schizophrenia.

## Introduction

Schizophrenia is a severe psychiatric disorder characterized by positive (e.g., delusions and hallucinations) and negative (e.g., amotivation, asociality) symptoms experienced by patients. This psychopathology is further associated with significant deficits in multiple cognitive domains including attention, executive functions, social cognition, processing speed, verbal and visual memory, as well as working memory^1–3^. Cognitive deficits have been observed early in the course of the illness, with cognitive scores of first-episode psychosis patients ranging from 1.0 to 1.5 standard deviations below the population average^3,4^. Importantly, there is vast literature establishing cognitive deficits to be stronger predictors of social and occupational dysfunction in schizophrenia than positive and negative symptoms^5,6^. Of these impairments, episodic memory deficits are among the most prominent and consequential for the functioning of individuals with schizophrenia^3,5^. This raises the need to better understand the pathophysiology of episodic memory deficits in schizophrenia.

The hippocampus is crucial for encoding and retrieving the details (e.g., context) of personal events^7^. In patients with schizophrenia, evidence from meta-analyses and literature reviews suggest significant hippocampal volume reduction when compared to healthy subjects^8–11^. The hippocampus is thought to be a crucial biomarker in the pathophysiology of schizophrenia^12^, given that structural deficits in this region (i) have been observed in the first episode of psychosis^13,14^, (ii) appear to predict progression to psychosis in individuals clinically at-risk^15^, and (iii) seem to further deteriorate after illness onset^16,17^. Noteworthy, several structural neuroimaging studies have shown volume reductions in the hippocampus to be associated with decreased episodic memory performance in schizophrenia^18,19^.

Hippocampal deficits in individuals with schizophrenia have also been observed in functional neuroimaging studies during resting-state activity^20^, emotional perception, and experience^21,22^, facial emotion processing^23^, as well as during episodic memory tasks^24–29^. Although less extensively studied, hippocampus-based functional connectivity studies have demonstrated abnormalities in schizophrenia patients when compared to healthy subjects. For instance, resting-state connectivity alterations were observed between the hippocampus and regions of the default mode network (DMN), including the medial prefrontal cortex, PCC, and the precuneus^30–35^. Moreover, during working memory tasks, connectivity alterations have been observed in patients with schizophrenia between the hippocampus and the PCC^36,37^, and executive regions such as the dorsolateral prefrontal cortex^38^ and the inferior frontal gyrus^39^. To our knowledge, no study has employed an episodic memory task to examine the connectivity patterns of hippocampus subregions in patients with schizophrenia.

In the past decades, evidence from rodents, primates, and humans has supported the segmentation of the hippocampus along the longitudinal axis (i.e., anterior–posterior, ventral–dorsal, and temporal–septal). For instance, it has been shown that these two sub-regions differ substantially in their anatomical and functional connections. In fact, results suggest that the anterior subregion is preferentially connected to the perirhinal cortex, the lateral temporal cortex extending to the temporal pole, the amygdala, striatum, the pre-supplementary motor area, while the posterior portion is preferentially connected to the posterior parahippocampal cortex, the pregenual anterior and posterior cingulate cortices, precuneus, thalamus, inferior parietal lobe, and the cuneus/lingual gyrus^40–45^. However, differences in functional connectivity between both subregions with frontal regions (e.g., vmPFC and dmPFC) remain unclear^40–42,44–46^. These hippocampal subregions have long been suggested to be involved in memory encoding and retrieval respectively^47,48^. As the posterior hippocampus may also be implicated in encoding^43,49–51^, the evidence in support of this dichotomy remains equivocal.

Only a few studies have examined the functional connectivity of hippocampal subregions in schizophrenia. In resting-state connectivity, the investigations performed thus far have shown deficits in functional connectivity mainly between the anterior hippocampus (aHippocampus) and DMN regions, such as the medial prefrontal cortex and the PCC^31,35,52,53^, as well as between the posterior hippocampus (pHippocampus) and the anterior cingulate cortex^31,35,52,53^. Alterations of connectivity between the aHippocampus and temporal regions^35,53,54^, as well as between the pHippocampus and the dorsolateral prefrontal cortex^54^, have also been observed, though less consistently. To our knowledge, only one task-based functional connectivity study has been published on hippocampal subregions in schizophrenia showing reduced connectivity from the posterior hippocampus to the inferior frontal gyrus during a complex *working* memory task^39^. It is striking that no research on functional connectivity of hippocampal subregions has been performed in schizophrenia using an *episodic* memory task, despite the hippocampus being well-known to play a key role in this cognitive domain ^7^.

In view of the extant literature, the primary objective of the current study was to examine the functional connectivity of hippocampal subregions in schizophrenia during an episodic memory task. As a secondary objective, we sought to replicate the structural deficits that have been reported in these subregions in schizophrenia^55–59^. Finally, given that emotional experience may alter episodic memory performance in schizophrenia^60^ and that the aHippocampus is more strongly connected to the limbic system than the posterior hippocampus^43^, we employed an episodic memory task with an emotional component in order to explore potential interactions between cognition and emotion.

## Results

### Sociodemographic, clinical, and task performance

Healthy controls (HC) and schizophrenia patients (SZ) subjects did not differ in terms of age or gender. The SZ group attained significantly lower scores on the bloc design subset of the WAIS-III than the HC group, as well as SZ, reported lower education levels than HC (Table 1). Finally, a poorer performance was observed in SZ participants in memory accuracy during the retrieval condition of the functional magnetic resonance imaging (fMRI) task, across negative, positive, and neutral images (approximatively 25% difference between groups). Clinical characteristics of individuals with SZ can be found in Table 1. Furthermore, a significant between-group difference was observed on a number of removed volumes during the encoding task (*p* = 0.02) but not during retrieval conditions (*p* > 0.05).

**Table 1:** Sociodemographic, clinical, and task performance characteristics of the sample.

Variables	Healthy controls (*n* = 45)	Schizophrenia patients (*n* = 41)	Statistics	*p* Value
*Sociodemographic and clinical*				
Age (mean, SD)	30.16 (8.63)	32.15 (6.89)	1.38	0.243
Sex (male %)	46.70%	51.20%	0.178	0.673
Block design (WAIS-III)	11.79 (3.18)	9.14 (3.15)	12.71	0.001
Education level (in yr)	17.78 (3.96)	11.56 (3.02)	65.96	<0.001
Age of onset	–	22.45 (5.54)	–	–
Medication—CPZ equiv. (in mg)	–	576.02 (358.99)	–	–
*PANSS*				
Positive symptoms	–	18.68 (6.74)	–	–
Negative symptoms	–	20.51 (6.92)	–	–
General symptoms	–	40.81 (9.89)	–	–
*Task performance*				
Memory accuracy				
Negative images	83.40%	63.33%	20.15	<0.001
Positive images	85.02%	58.70%	37.4	<0.001
Neutral images	77.79%	51.30%	43.88	<0.001

*Note*. On continuous measures, standard deviations are presented in parentheses. The difference between groups on continuous variables was tested using one-way ANOVA (*F*-statistic is reported) and categorical variables with chi-square test (*χ*^2^ is reported). *CPZ* chlorpromazine, *SD* standard deviation.

### Weighted seed-based connectivity analyses

First, when examining the connectivity patterns of the anterior and posterior hippocampus between HC and SZ during the retrieval condition (e.g., across a block of Run-1) compared to the encoding condition (e.g., across a block of Run-2) (i.e., Diagnosis [HC versus SZ]*Condition [Retrieval versus Encoding]*Seeds [Hemisphere specific anterior versus posterior hippocampus] interaction], no significant difference was observed. We further examined the different connectivity patterns of the anterior and posterior hippocampus between HC and SZ (i.e., Diagnosis*Seeds) interaction within each condition separately.

**Fig. 1:**
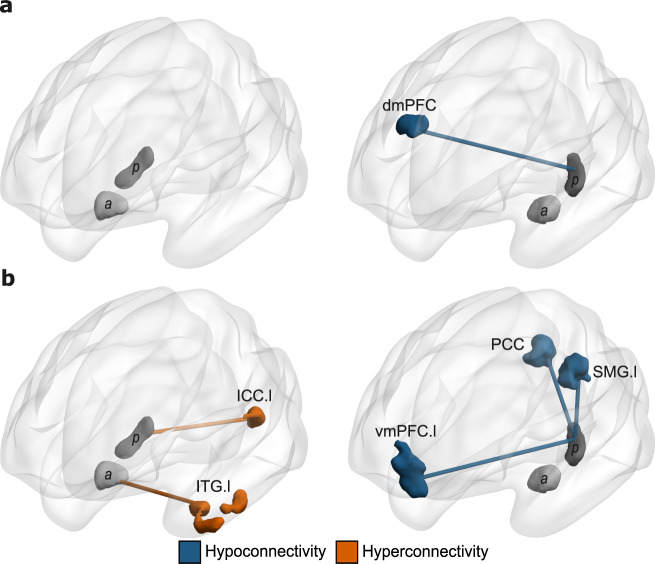
Brain regions (targets) showing significant between-group differences in connectivity with hippocampal subregions from the diagnosis-by-seeds interactions. **a** Results from the diagnosis-by-seeds of the left hemisphere in the encoding condition. **b** Results from the diagnosis-by-seeds in the retrieval condition; blue connections/regions indicate significant hypoconnectivity with the target; orange connections/regions indicate significant hyperconnectivity with the target. a anterior hippocampus, p posterior hippocampus, vmPFC ventromedial prefrontal cortex, ICC intracalcarine cortex, dmPFC dorsomedial prefrontal cortex, PCC posterior cingulate cortex, SMG supramarginal gyrus, ITG inferior temporal gyrus, L/l left, R/r right.

**Fig. 2:**
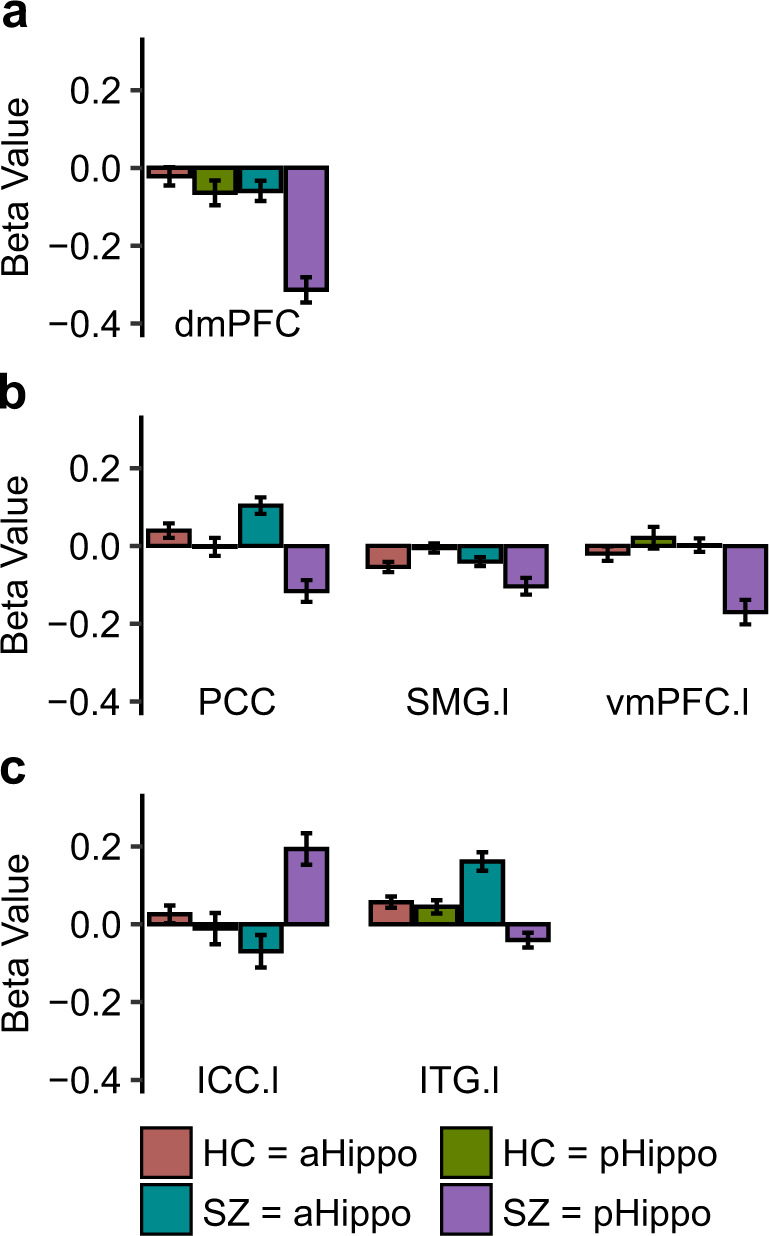
Beta coefficients of significant diagnosis by seeds connectivity results. **a** Results from the interaction diagnosis-by-seeds of the left hemisphere in the encoding condition. Hippocampus-dmPFC connectivity (beta values from left to right: −0.02, −0.07, −0.06, −0.31); **b** Results from the interaction diagnosis-by-seeds of the left hemisphere in the retrieval condition, hippocampus-PCC connectivity (beta values: 0.04, −0.002, 0.10, −0.12), hippocampus-SMG connectivity (beta values: −0.05, −0.005, −0.04, −0.10), Hippocampus-vmPFC connectivity (beta values: −0.02, 0.02, 0.002, −0.17); **c** Results from the interaction diagnosis-by-seeds of the right hemisphere in the retrieval condition. Hippocampus-ICC connectivity (beta values: 0.03, −0.01, −0.07, 0.19), hippocampus-ITG connectivity (beta values: 0.06, 0.04, 0.16, −0.04). HC healthy controls, SZ schizophrenia patients, aHippo anterior hippocampus, pHippo posterior hippocampus, vmPFC ventromedial prefrontal cortex, ICC intracalcarine cortex, dmPFC dorsomedial prefrontal cortex, PCC posterior cingulate cortex, SMG supramarginal gyrus, ITG inferior temporal gyrus, l left, r right. Error bars represent the standard error of the mean (s.e.m.).

**Fig. 3:**
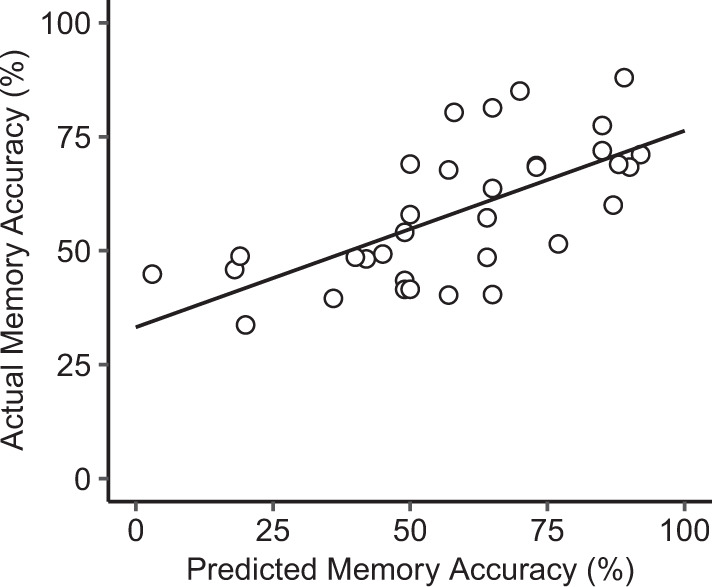
Scatter plot and regression line of the actual by predicted values of multiple regression analysis on memory accuracy performance in patients with schizophrenia. Available data was available for *n* = 35 patients (85.4% of the total patient sample).

In the Encoding condition, the Diagnosis by Left Hemisphere Seeds (aHippocampus versus pHippocampus) revealed significant connectivity differences with the intracalcarine cortex (ICC) and the dorsomedial prefrontal cortex (dmPFC). However, adding the number of removed volumes (motion scrubbing) as covariate only replicated the dmPFC (See Table 2, Figs. 1, 2A, and 3). Adding the subject’s average of Artifact Detection Tool (ART’s) composite motion measure on the remaining volumes as covariate yield similar results (Supplementary Table 2). Post hoc analyses showed reduced pHippocampus–dmPFC connectivity (*F*_1,84_ = 30.00, *p* < 0.001), in SZ compared to HC. In within-group comparisons, we observed that pHippocampus–dmPFC connectivity was significantly reduced compared to aHippocampus–dmPFC in SZ (*p* < 0.001), but not in HC (*p* = 0.160). No significant target was observed for the diagnosis by right hemisphere seeds (anterior versus posterior subregions) during Encoding.

**Table 2:** Brain regions demonstrating significant connectivity patterns between anterior and posterior Hippocampus in patients versus healthy controls.

Regions	MNI coordinates of peak				p-FDR	Voxels
	*Left/Right*	*x*	*y*	*z*		
*Within encoding conditions*						
Diagnosis*Seeds (left)						
dmPFC	R	6	48	26	0.04	169
Diagnosis*Seeds (right)						
n.s.						
*Within retrieval conditions*						
Diagnosis*Seeds (left)						
Precuneus/PCC	–	0	−50	44	<0.01	369
vmPFC	L	−6	60	−14	0.013	193
SMG^A^	L	−32	−38	34	0.013	185
Diagnosis*Seeds (right)						
ITG/temporal^B^	L	−36	−10	−28	<0.01	545
Intralcalcarine cortex	L	−12	−84	10	<0.01	221

*Note*. ^A^ = cluster that included mainly the supramarginal gyrus and some voxels encompassing the inferior parietal lobule excluding the SMG; ^B^ = cluster that included mainly the inferior temporal gyrus and some voxels encompassing the fusiform gyrus, the anterior Parahippocampal gyrus and the uncus. *dmPFC* dorsomedial prefrontal cortex, *PCC* posterior cingulate cortex, *vmPFC* ventromedial prefrontal cortex, *SMG* supramarginal gyrus, *ITG* inferior temporal gyrus, *n.s.* not significant.

In the Retrieval condition, the diagnosis by left hemisphere seeds interaction resulted in significant connectivity differences in the precuneus/posterior cingulate cortex (PCC), the ventromedial prefrontal cortex (vmPFC), and a cluster that includes the supramarginal gyrus (SMG) and voxels spanning the inferior parietal lobule (see Table 2 and Fig. 1). Adding the subject’s average of ART’s composite motion measure on the remaining volumes as covariate replicated these results (Supplementary Table 2). Post hoc analyses revealed decreased connectivity between the left pHippocampus with the precuneus/PCC (*F*_1,84_ = 10.06, *p* < 0.001), while weaker increased connectivity was observed between the left aHippocampus and the precuneus/PCC (*F*_1,84_ = 5.23, *p* = 0.025), in SZ compared to HC (see Fig. 2B). Moreover, we observed that the pHippocampus–vmPFC connectivity (*F*_1,84_ = 20.69, *p* < 0.001) and the pHippocampus-SMG (*F*_1,84_ = 16.48, *p* < 0.001) were reduced in SZ compared to HC (see Fig. 2).

Diagnosis by right hemisphere seeds during the retrieval condition suggested significant connectivity differences with the inferior temporal gyrus (ITG) and the ICC (see Table 2, Figs. 1 and 2C). These differences were also observed after adding the subject’s average of ART’s composite motion measure on the remaining volumes as covariate (Supplementary Table 2). Post hoc analyses revealed increased connectivity between the aHippocampus and the ITG (*F*_1,84_ = 14.57, *p* < 0.001) in SZ compared to HC. Furthermore, increased connectivity between the pHippocampus and the ICC (*F*_1,84_ = 12.94, *p* = 0.001) was observed in SZ compared to HC. Within-group comparisons suggested that aHippocampus–ITG connectivity was significantly greater than pHippocampus–ITG in SZ (*p* < 0.001), but not in HC (*p* = 0.456) and that pHippocampus–ICC connectivity was greater than aHippocampus–ICC in SZ (*p* < 0.001), but not in HC (*p* = 0.412).

Finally, the diagnosis by stimuli valence (i.e., POS, NEG, and NEU) interaction analysis revealed that none of the main connectivity findings were significantly altered by stimuli valence. Correlational analyses suggested that no clinical variables, including the level to antipsychotic medication (i.e., chlorpromazine equivalents) were significantly associated with our connectivity results (*p* > 0.341).

### Voxel-based morphometry

We further examined GM volume deficits in each hippocampal subdivision and their functional connectivity targets. The GM volumes of the left anterior (*xyz* = −30,−18,−14; threshold-free cluster enhancement (TFCE)-small volume correction (SVC) < 0.001) and posterior Hippocampus (*xyz* = −24,−42,2; TFCE-SVC < 0.001), as well as the right anterior (*xyz* = 24,−21,−12; TFCE-SVC = 0.003) and posterior Hippocampus (*xyz* = 21,−39,6, TFCE-SVC < 0.001) were significantly less in SZ patients compared to HC. No statistically significant between-group differences in GM volume were observed for connectivity targets of hippocampal subregions.

### Neurobiological correlates of memory accuracy in patients with schizophrenia

Bivariate Pearson correlations revealed that in the SZ group, the aHippocampus–ITG and the pHippocampus–ICC connectivity, observed in the retrieval condition, were significantly associated with memory accuracy during the retrieval condition (*r* = 0.348, *p* = 0.03 and *r* = −0.470, *p* = 0.003, respectively). These relationships were not statistically significant in healthy subjects (*r* = −0.002 and *r* = −0.114, respectively). Moreover, the block design subset of the WAIS (*r* = 0.425, *p* = 0.014; available data for 35 patients) and education level (*r* = 0.345, *p* = 0.031) was also significantly associated with memory accuracy. No other significant relationship with memory accuracy was observed (e.g., GM volume of hippocampal subregions, medication, and clinical symptomatology).

Forward stepwise multiple regression revealed a final model suggesting that the aHippocampus–ITG and pHippocampus–ICC connectivity remained statistically independent predictors of memory accuracy (*β* = 0.354, *p* = 0.021 and *β* = 0.437, *p* = 0.005, respectively), while the block design subset of the WAIS was no longer statistically significant (*β* = 0.295, *p* = 0.052). Education level was not included in the final selected model by the stepwise regression. These three variables factors showed low collinearity (VIF < 2). Furthermore, the model explained 37.2% of the variance (adjusted R-squared) associated with memory accuracy in patients with schizophrenia.

## Discussion

In the current study, we investigated the functional connectivity of distinct subregions of the hippocampus in patients with schizophrenia during an episodic memory task. We observed decreased pHippocampus–dmPFC connectivity in schizophrenia patients, relative to controls, during the encoding condition. Furthermore, we found reduced connectivity between the pHippocampus and DMN regions including the precuneus, the SMG, and the vmPFC, but increased pHippocampus–ICC connectivity in participants with schizophrenia during the retrieval condition. We also observed increased connectivity between the aHippocampus and anterior temporal regions in patients with schizophrenia. The altered pHippocampus–ICC and aHippocampus–ITG connectivity was correlated with memory accuracy. The functional connectivity and behavioral results were complemented by the VBM analysis, which revealed reduced GM volume in the bilateral anterior and posterior hippocampus in patients versus healthy controls.

Although the number of studies investigating the functional connectivity of the hippocampal subregions in patients with schizophrenia is limited, similar results were observed by researchers using resting-state paradigms. In fact, some authors have found that patients showed disrupted functional connectivity between the aHippocampus and anterior temporal regions^35,52,53^. The former connectivity is particularly relevant since these anterior medial temporal lobe structures are known to play an important role in episodic memory^61–63^. In contrast to what we observed, the aforementioned studies found that only the anterior portion of the hippocampus was significantly disconnected from the precuneus/PCC and the vmPFC^35,52,53^. A potential explanation for this discrepancy could be that we examined functional connectivity as related to episodic memory, while previous studies examined connectivity at rest. It is also worth mentioning that our results are congruent with structural and functional neuroimaging literature in healthy volunteers which consistently reported that the posterior subregion of the hippocampus is preferentially connected (posterior > anterior) to the posterior parahippocampal gyrus as well as DMN regions, while the anterior part is preferentially associated (anterior > posterior) with the perirhinal cortex, amygdala, and extended temporal pole^40–45^. This is further supported by studies using meta-analytical connectivity modeling and a reverse inference approach which suggest a clear Emotion (e.g., face monitor/discrimination, emotional valence, threat, anxiety, fear, and neutral stimuli and faces) to Cognition (e.g., world-centric behaviors including navigation, perceptual functioning, and processing information in its environmental context) gradient along the longitudinal axis of the hippocampus (i.e., anterior–posterior axis) ^50,51^.

Although it has long been assumed that anterior–posterior subregions may play specific roles in encoding and retrieval^47,64,65^, this dichotomy is still a subject of debate^50,51,61,66^. Following recent results from the BrainMap and NeuroSynth databases that suggest equally distributed encoding/retrieval processes along the hippocampal axis^50^, our results support the importance of the pHippocampus in both the encoding and retrieval conditions as well as the aHippocampal subregions during retrieval condition. Indeed, in patients with schizophrenia, deficits in connectivity between pHippocampus and anterior DMN region has been observed during the encoding condition while the pHippocampus was altered mostly with posterior DMN during retrieval condition. Past evidence suggests that the posterior DMN regions are engaged in processes supporting successful episodic retrieval but are deactivated during encoding conditions^67–70^. This phenomenon, also called the encoding-retrieval flip pattern^67^, is however less clear for anterior DMN regions (i.e., vmPFC and dmPFC)^68,71–73^. Nonetheless, our results indicate that patients with schizophrenia exhibit deficits in connectivity between the pHippocampus and posterior DMN regions that may reflect failure in the encoding-retrieval flip pattern.

Interestingly, we observed a significant increased pHippocampus–ICC connectivity during retrieval condition and this was found to be strongly associated with memory accuracy in schizophrenia. It is worth mentioning that connectivity alterations between hippocampal subregions and ICC have also been observed by other authors^31,54^. The ICC, including the primary visual cortex, is known to send projections to posterior parietal regions through the dorsal visual processing stream^74–76^. The posterior parahippocampal gyrus thus receives inputs from these posterior parietal regions (i.e., mainly caudal inferior parietal lobule) which, in turn, projects to the pHippocampus^77–79^, justifying the role of both structures in integrating contextual information of a stimuli as well as in retrieving the initial encoding context^80,81^. The alteration between the pHippocampus–ICC is coherent with a growing body of literature showing that the activity and connectivity of occipital regions involved in the early processing of visual stimuli are impaired in schizophrenia^82,83^.

Our study has a few limitations that need to be acknowledged. First, although the sample of the current study was sufficiently powered to detect differences in functional connectivity between patients and controls during episodic memory, it may have not been sufficiently powered to examine the interaction between emotional valence and memory conditions. We also acknowledge that some of our findings (or lack of findings) may be explained by the specificities of the episodic memory task that we used. Indeed, we used an incidental encoding task whereby participants were not explicitly asked to memorize the information that was presented to them in the first run. Furthermore, the retrieval condition consisted of a recognition memory condition, rather than a free recall condition. In both cases, this may explain why we did not observe impaired connectivity between hippocampal sub-regions and executive regions, such as the dlPFC. Another limitation has to do with the fact that the VBM was performed using ROIs and did not examine the whole brain. Finally, we acknowledge that antipsychotic treatment may have influenced results. Previous trials have shown that some second-generation antipsychotics produce small beneficial effects on episodic memory in schizophrenia^84^. At the neural level, the effects of antipsychotics on hippocampal volumes have, however, been inconsistent and mixed^85^. Although preliminary, a few fMRI studies have shown that second-generation antipsychotics may normalize hippocampal activity and connectivity during episodic memory in schizophrenia^86,87^. It remains unclear, however, how these latter observations may relate to our own findings considering that patients were treated with second-generation antipsychotics, and displayed hippocampal dysconnectivity nevertheless. Moreover, we found no significant association between antipsychotic dosage (e.g., chlorpromazine equivalents) and hippocampal dysconnectivity.

In the current study, we examined the functional connectivity of hippocampal subregions performed in schizophrenia using an *episodic* memory task. We observed a complex pattern of connectivity alterations from the anterior and posterior hippocampal subregions to the default-mode, temporal and occipital regions. Our results highlight the importance of the hippocampal subregions, which are known to play a crucial role in the rodent model of psychosis^88^ and the pathophysiology of schizophrenia^12^. In the future, longitudinal fMRI studies will need to be performed in order to understand the role of hippocampal subregions in the psychosis spectrum, by pursuing research in individuals a high risk for psychosis, in patients with the first episode of psychosis, as well as schizophrenia patients who are treatment-resistant. Investigations in drug-free patients, as well as in unaffected first-degree relatives are also warranted. Attention will also need to be paid to task design characteristics.

## Methods

### Sample

Forty-one individuals with schizophrenia (SZ) in a stable state (i.e., no psychotic relapse within the last 2 months and no change in their antipsychotic medication within the month preceding the study) and 45 healthy controls (HC) were recruited for the purposes of this study. Schizophrenia was diagnosed according to DSM-IV-TR^88^ using the *Structured Clinical Interview for DSM-IV* (SCID), and participants were all treated with second-generation antipsychotics. Urine drug screenings were performed. Psychiatric symptoms were evaluated with the *Positive and Negative Syndrome Scale* (PANSS^89^), while IQ was estimated based on the vocabulary, similarities, and block design subtests of the *Wechsler abbreviated scale of intelligence* (WASI^90^). Antipsychotic dosage was calculated using chlorpromazine equivalents^91,92^. Control participants were screened using the non-patient edition of the SCID in order to rule out any Axis-I psychiatric disorders. General exclusion criteria were age younger than 18 years or older than 45 years, past or present neurological disorder, substance use disorder (during the year preceding the study for participants; lifetime for controls), or contraindications for magnetic resonance imaging (MRI) (e.g., cardiac pacemaker, aneurysm clip).

The ethics committees of the *Centre de recherche de l’Institut en Santé Mentale de Montréal* and the *Regroupement de Neuroimagerie du Québec* approved the study protocol. In agreement with the Declaration of Helsinki, we obtained written informed consent from participants before the experiment. The ability of participants with SZ to give informed consent was established using the guidelines of the *Canadian Psychiatric Association*. The data are not publicly available as they contain information that could compromise research participant privacy/consent.

### Experimental procedure

In the first run, participants passively viewed blocks of pictures while in the MRI machine. The stimuli were selected from the *International Affective Picture System* (IAPS)^93^ based on normative valence and were matched for content (e.g., people, animals, and landscapes). The images presented differed in their valence, with each image category being shown in separate blocks lasting 48.5 s, resulting in three experimental conditions: positive (POS), negative (NEG), and Neutral (NEU) content. Although subjects were aware that a memory task would follow the encoding-processing run, they were not explicitly instructed to remember the images. Instead, to ensure that participants were attentive to the images presented during encoding, they were asked to indicate with the press of a button whether they saw a person or part of a person in the picture. This task served as an incidental learning procedure, which allowed for secondary analyses of potential interactions between learning (i.e., encoding) and emotion. This encoding task was then followed by an unrelated mental rotation task lasting 15 min as a means of separating both incidental encoding and subsequent recognition memory.

The retrieval portion consisted of viewing 48.5 s blocks of emotionally positive, negative, and neutral pictures similar to the incidental encoding task. During this second run, however, 50% of the stimuli in each block originated from the encoding task (previously viewed), while 50% were novel (never viewed before). The order of presentation of stimuli was randomized. There were 16-s periods of rest separating the blocks from one another. Each block contained ten images and each block type was repeated four times. Each picture appeared for 3000 ms followed by a blank screen with a fixation point for an average of 1.75 s (ranging from 1 to 2.5 s and giving an average interstimulus interval (ISI) of 4.75 s). During this recognition memory task, participants were to determine, by pressing the correct button, which of the stimuli were old and which were new. This task allowed for primary analyses on recognition memory regardless of emotional valence, and secondary analyses on potential interactions between retrieval and emotion.

### MRI acquisition parameters

Blood oxygen level-dependent (BOLD) data were acquired using a T2-weighted gradient echo-planar imaging (EPI) sequence [repetition time (TR) = 3000 ms, echo time (TE) = 30 ms, flip angle = 90°, matrix 64 × 64; voxel size = 3.5 mm^3^; 41 axial slices] on a 3.0 Tesla TRIO-TIM MRI system. The functional slices were angled parallel to the AC–PC line. An inline retrospective motion correction algorithm was employed while the EPI images were acquired. Individual high-resolution co-planar anatomical images were also acquired using a three-dimensional, spoiled gradient-echo sequence (TR = 19 ms; TE = 4.92 ms; FA = 25^o^; matrix size: 256 × 256; voxel size; 1 mm^3^; 176 sagittal slices).

### fMRI data preprocessing

Functional images were realigned, corrected for motion artifacts with the Artifact Detection Tool^94^ (ART, setting a threshold of 0.9 mm subject ART’s composite motion and a global signal threshold of *Z* = 5) with the implemented in CONN Toolbox^95^, high-passes filtered (>0.008 Hz) and co-registered to the corresponding anatomical image. The anatomical images were segmented (into GM, white matter, and cerebrospinal fluid) and normalized to the Montreal Neurological Institute (MNI) stereotaxic space. Functional images were then normalized based on structural data, spatially smoothed with a 6 mm full-width-at-half-maximum (FWHM) 3D isotropic Gaussian kernel, and resampled to 2 mm^3^ voxels. For the preprocessing, the anatomical component-based noise correction method (aCompCor strategy^96^), was employed to remove confounding effects from the BOLD time series, such as the physiological noise originating from the white matter and cerebrospinal fluid. This method was found to increase the validity and sensitivity of analyses^97^.

### Weighted seed-based connectivity analyses

Hippocampus subregions in MNI space were constructed using maximum probability maps through the Anatomy Toolbox^98^. Voxels were included in the hippocampus (also comprising CA1–4 and the dentate gyrus^99^) if their likelihood of being located in the hippocampus was at least 60%. Voxels were excluded if they had any probability of belonging to the amygdala. These steps were taken in order to prevent false positives due to the proximity of the aHippocampus and amygdala in functional connectivity/GM volume differences. The hippocampus maps were then binarized and sectioned into anterior (*y* = −11 to −21) and posterior (*y* = −32 to −43) subregions along the *y*-axis, with a gap (*y* = −22 to −31), to avoid any overlap^40^.

Physiological noise, realignment parameters, and movement artifacts were regressed out as confounding effects from the BOLD time-series at each voxel. The main activation effects of the conditions were also regressed out to avoid spurious connectivity due to task co-activation. The residual time-series were weighted by the appropriate hemodynamic response function-convolved regressor to derive task condition-specific time-series for weighted functional connectivity analyses^95^. In the first-level analysis, weighted seed-based connectivity maps were calculated with a weighted least-squares linear model (bivariate regression) between the time-course of each region-of-interest (ROI) seed-to-voxel, for each subject.

In the second-level analysis, between-group differences were investigated (SZ versus HC), thus allowing to search for connections that were altered in SZ. Since we were interested in examining differences in the distinct connectivity profiles of the anterior and posterior hippocampus^40^ between the two groups and task conditions, we performed a group (HC versus SZ) by task (Encoding versus Retrieval) by seed (aHippocampus versus pHippocampus, each hemisphere separately) interaction analysis. A two-sample *t* test was performed between groups on the number of removed volumes (motion scrubbing) during the task. Healthy subjects (M = 6.74, SD = 13.5) and SZ (M = 15.4, SD = 16.76) did differ significantly from each other (*t* = 2.63, *p* = 0.01). Therefore, the number of removed volumes was added as a covariate in the second-level analysis if the two-groups significantly differed (*p* < 0.05). Second-level analyses were also performed using the average of the ART composite motion measure on the remaining volumes (Supplementary Table 1). Since more than 88% of total volumes remained after motion scrubbing (>481/545 volumes), for every participant, no participant was excluded. The peak threshold was set at *p* < 0.005 (two-sided) with a cluster threshold corrected for the false discovery rate of *p* < 0.05.

Finally, regression coefficients of significant targets were extracted using MARSeille Boîte À Région d’Intérêt (MarsBaR) toolbox (sourceforge.net/projects/marsbar/). Within-group post hoc analyses were performed to assess the preferential connectivity targets between aHippocampus versus pHippocampus. In order to assess whether specific emotions may have driven functional connectivity results, we performed post hoc analyses using IBM SPSS Statistics 25 for Windows. Moreover, relationships between functional connectivity regression coefficients and clinical correlates were tested with Pearson’s correlation. Imaging results were visualized with BrainNet^100^, data were displayed using ggplot2^101^, and figures were built with Inkscape (inkscape.org).

### Voxel-based morphometry

Anatomical images were preprocessed using the *Computational Anatomy Toolbox* for SPM12 (CAT12)^102^. The CAT12 preprocessing pipeline includes image realignment and tissue segmentation into gray matter (GM), white matter, and cerebrospinal fluid. The segmented scans were normalized to a 1.5 mm isotropic predefined adult template provided by the CAT12 toolbox (in MNI space), using the Diffeomorphic Anatomic Registration Through Exponentiated Lie algebra algorithm (DARTEL). The deformation parameters were estimated using the nonlinear spatial registration of DARTEL and applied to the tissue segmentations. Modulation with the Jacobian determinant of the deformation was executed to preserve the total amount of GM signal following spatial normalization. The segmented and modulated GM images were then smoothed using a 6-mm FWHM Gaussian kernel.

Retrospective quality assurance was performed using a weighted image quality rating (IQR) based on noise, inhomogeneities, and image resolution, provided by the CAT12 toolbox. Individuals were excluded from further analysis if the IQR was at >2 standard deviations below the mean percent rating of the sample (M = 77.8%, SD = 5.94%). This resulted in the exclusion of three HC and four SZ participants.

In order to perform analysis on hippocampal subregions and their respective connectivity targets, the forward deformation DARTEL field of an ICBM152 T1 image (MNI space) was saved and applied to each binary masked ROI. Two-sample *t* tests were then performed on resulting images with total intracranial volume and IQR to remove their potential confounding effects on ROIs. Moreover, control over the family wise error rate through nonparametric permutation TFCE approach^103^ was performed with 5000 permutations within each ROIs separately (SVC). Standard parameter values *E* = ½, *H* = 2 were used for the TFCE method. Significant differences were detected at a *p* < 0.05 (TFCE-SVC). Relationships between GM volume of hippocampal subregions and clinical correlates were performed with Pearson’s correlations.

### Neurobiological correlates of memory accuracy in patients with schizophrenia

In order to better understand the correlates of memory accuracy, as defined as percentage images well identified, in patients with schizophrenia, bivariate Pearson’s correlations were first performed between neurobiological features (connectivity and VBM analyses) and memory accuracy during the retrieval condition. The statistically significant neurobiological factors were then entered in a multiple linear regression model. Clinical variables (e.g., medication, age of onset, clinical symptomatology, and IQ) associated with memory accuracy (*p* < 0.05) were entered as covariates in the multiple linear regression. A stepwise (forward selection) regression model was used to prevent overfitting.

### Reporting summary

Further information on research design is available in the Nature Research Reporting Summary linked to this article.

## Supplementary information

Supplementary Material

REPORTING SUMMARY

## Data Availability

The data are not publicly available as they contain information that could compromise research participant privacy/consent. The data that support the findings of this study are available upon reasonable request from the corresponding author. S.P. but are only redistributable to researchers engaged in IRB approved research collaborations.

## Code availability

Embedded pipelines in toolboxes were used to generate results with no custom code necessary for conclusions or replication: CONN Toolbox version 18b (https://web.conn-toolbox.org/) and CAT12 (http://www.neuro.uni-jena.de/cat/).

## References

[1] Nuechterlein KH (2004). Identification of separable cognitive factors in schizophrenia. Schizophr. Res..

[2] Savla GN, Vella L, Armstrong CC, Penn DL, Twamley EW (2013). Deficits in domains of social cognition in schizophrenia: a meta-analysis of the empirical evidence. Schizophr. Bull..

[3] Schaefer J, Giangrande E, Weinberger DR, Dickinson D (2013). The global cognitive impairment in schizophrenia: consistent over decades and around the world. Schizophr. Res..

[4] Keefe R (2014). Cognitive effects of pharmacotherapy for major depressive disorder: a systematic review. J. Clin. Psychiatry.

[5] Fett A-KJ, Viechtbauer W, Penn DL, van Os J, Krabbendam L (2011). The relationship between neurocognition and social cognition with functional outcomes in schizophrenia: a meta-analysis. Neurosci. Biobehav. Rev..

[6] Tolman AW, Kurtz MM (2012). Neurocognitive predictors of objective and subjective quality of life in individuals with schizophrenia: a meta-analytic investigation. Schizophr. Bull..

[7] Tulving E, Markowitsch HJ (1998). Episodic and declarative memory: role of the hippocampus. Hippocampus.

[8] Honea R, Crow TJ, Passingham D, Mackay CE (2005). Regional deficits in brain volume in schizophrenia: a meta-analysis of voxel-based morphometry studies. Am. J. Psychiatry.

[9] Nakahara S, Matsumoto M, van Erp TG (2018). Hippocampal subregion abnormalities in schizophrenia: a systematic review of structural and physiological imaging studies. Neuropsychopharmacol. Rep..

[10] Nelson MD, Saykin AJ, Flashman LA, Riordan HJ (1998). Hippocampal volume reduction in schizophrenia as assessed by magnetic resonance imaging: a meta-analytic study. Arch. Gen. Psychiatry.

[11] Steen RG, Mull C, Mcclure R, Hamer RM, Lieberman JA (2006). Brain volume in first-episode schizophrenia: systematic review and meta-analysis of magnetic resonance imaging studies. Br. J. Psychiatry.

[12] Lieberman J (2018). Hippocampal dysfunction in the pathophysiology of schizophrenia: a selective review and hypothesis for early detection and intervention. Mol. Psychiatry.

[13] Adriano F, Caltagirone C, Spalletta G (2012). Hippocampal volume reduction in first-episode and chronic schizophrenia: a review and meta-analysis. Neuroscientist.

[14] Vita A, De Peri L (2007). Hippocampal and amygdala volume reductions in first-episode schizophrenia. Br. J. Psychiatry.

[15] Schobel SA (2009). Differential targeting of the CA1 subfield of the hippocampal formation by schizophrenia and related psychotic disorders. Arch. Gen. Psychiatry.

[16] Ho NF (2017). Progression from selective to general involvement of hippocampal subfields in schizophrenia. Mol. Psychiatry.

[17] Pujol N (2014). Hippocampal abnormalities and age in chronic schizophrenia: morphometric study across the adult lifespan. Br. J. Psychiatry.

[18] Antoniades M (2018). Verbal learning and hippocampal dysfunction in schizophrenia: a meta-analysis. Neurosci. Biobehav. Rev..

[19] Duan X (2020). Reduced hippocampal volume and its relationship with verbal memory and negative symptoms in treatment-naive first-episode adolescent-onset schizophrenia. Schizophr. Bull.

[20] Kühn S, Gallinat J (2013). Resting-state brain activity in schizophrenia and major depression: a quantitative meta-analysis. Schizophr. Bull..

[21] Dugré JR, Bitar N, Dumais A, Potvin S (2019). Limbic hyperactivity in response to emotionally neutral stimuli in schizophrenia: a neuroimaging meta-analysis of the hypervigilant mind. Am. J. Psychiatry.

[22] Taylor SF (2012). Meta-analysis of functional neuroimaging studies of emotion perception and experience in schizophrenia. Biol. Psychiatry.

[23] Li H, Chan RC, McAlonan GM, Gong Q-Y (2010). Facial emotion processing in schizophrenia: a meta-analysis of functional neuroimaging data. Schizophr. Bull..

[24] Achim AM, Lepage M (2005). Episodic memory-related activation in schizophrenia: meta-analysis. Br. J. Psychiatry.

[25] Francis MM (2016). Functional neuroanatomical correlates of episodic memory impairment in early phase psychosis. Brain Imaging Behav..

[26] Heckers S (2001). Neuroimaging studies of the hippocampus in schizophrenia. Hippocampus.

[27] Leavitt VM, Goldberg TE (2009). Episodic memory in schizophrenia. Neuropsychol. Rev..

[28] Öngür D (2006). The neural basis of relational memory deficits in schizophrenia. Arch. Gen. Psychiatry.

[29] Ragland JD (2015). Functional and neuroanatomic specificity of episodic memory dysfunction in schizophrenia: a functional magnetic resonance imaging study of the relational and item-specific encoding task. JAMA Psychiatry.

[30] Kraguljac NV, White DM, Hadley J, Reid MA, Lahti AC (2014). Hippocampal‐parietal dysconnectivity and glutamate abnormalities in unmedicated patients with schizophrenia. Hippocampus.

[31] Kraguljac NV (2016). Aberrant hippocampal connectivity in unmedicated patients with schizophrenia and effects of antipsychotic medication: a longitudinal resting state functional MRI study. Schizophr. Bull..

[32] Li S (2019). Dysconnectivity of multiple brain networks in schizophrenia: a meta-analysis of resting-state functional connectivity. Front. Psychiatry.

[33] Sommer IE, Clos M, Meijering AL, Diederen KM, Eickhoff SB (2012). Resting state functional connectivity in patients with chronic hallucinations. PloS ONE.

[34] Zhang Y (2019). Association between NRGN gene polymorphism and resting-state hippocampal functional connectivity in schizophrenia. BMC Psychiatry.

[35] Zhou Y (2008). Altered resting-state functional connectivity and anatomical connectivity of hippocampus in schizophrenia. Schizophrenia Res..

[36] Meda SA, Stevens MC, Folley BS, Calhoun VD, Pearlson GD (2009). Evidence for anomalous network connectivity during working memory encoding in schizophrenia: an ICA based analysis. PloS ONE.

[37] Wolf RC (2009). Temporally anticorrelated brain networks during working memory performance reveal aberrant prefrontal and hippocampal connectivity in patients with schizophrenia. Prog. Neuropsychopharmacol. Biol. Psychiatry.

[38] Meyer-Lindenberg AS (2005). Regionally specific disturbance of dorsolateral prefrontal–hippocampal functional connectivity in schizophrenia. Arch. Gen. Psychiatry.

[39] Benetti S (2009). Functional integration between the posterior hippocampus and prefrontal cortex is impaired in both first episode schizophrenia and the at risk mental state. Brain.

[40] Chen AC, Etkin A (2013). Hippocampal network connectivity and activation differentiates post-traumatic stress disorder from generalized anxiety disorder. Neuropsychopharmacology.

[41] Libby LA, Ekstrom AD, Ragland JD, Ranganath C (2012). Differential connectivity of perirhinal and parahippocampal cortices within human hippocampal subregions revealed by high-resolution functional imaging. J. Neurosci..

[42] Persson J, Söderlund H (2015). Hippocampal hemispheric and long‐axis differentiation of stimulus content during episodic memory encoding and retrieval: an activation likelihood estimation meta‐analysis. Hippocampus.

[43] Poppenk J, Evensmoen HR, Moscovitch M, Nadel L (2013). Long-axis specialization of the human hippocampus. Trends Cogn. Sci..

[44] Poppenk J, Moscovitch M (2011). A hippocampal marker of recollection memory ability among healthy young adults: contributions of posterior and anterior segments. Neuron.

[45] Wagner G (2016). Resting state functional connectivity of the hippocampus along the anterior–posterior axis and its association with glutamatergic metabolism. Cortex.

[46] Qin S (2016). Large-scale intrinsic functional network organization along the long axis of the human medial temporal lobe. Brain Struct. Funct..

[47] Lepage M, Habib R, Tulving E (1998). Hippocampal PET activations of memory encoding and retrieval: the HIPER model. Hippocampus.

[48] Moser M-B, Moser EI (1998). Distributed encoding and retrieval of spatial memory in the hippocampus. J. Neurosci..

[49] Chase HW (2015). Evidence for an anterior–posterior differentiation in the human hippocampal formation revealed by meta-analytic parcellation of fMRI coordinate maps: focus on the subiculum. NeuroImage.

[50] Plachti A (2019). Multimodal parcellations and extensive behavioral profiling tackling the hippocampus gradient. Cereb. Cortex.

[51] Robinson JL (2015). Neurofunctional topography of the human hippocampus. Hum. Brain Mapp..

[52] Blessing EM (2020). Anterior hippocampal–cortical functional connectivity distinguishes antipsychotic naïve first-episode psychosis patients from controls and may predict response to second-generation antipsychotic treatment. Schizophr. Bull..

[53] Samudra N (2015). Alterations in hippocampal connectivity across the psychosis dimension. Psychiatry Res..

[54] Jiang Y (2019). Common increased hippocampal volume but specific changes in functional connectivity in schizophrenia patients in remission and non-remission following electroconvulsive therapy: a preliminary study. NeuroImage.

[55] Herold CJ (2013). Hippocampal volume reduction and autobiographical memory deficits in chronic schizophrenia. Psychiatry Res..

[56] Kalmady SV (2017). Clinical correlates of hippocampus volume and shape in antipsychotic-naïve schizophrenia. Psychiatry Res..

[57] McHugo M (2018). Regionally specific volume deficits along the hippocampal long axis in early and chronic psychosis. NeuroImage.

[58] Schobel SA (2009). Anterior hippocampal and orbitofrontal cortical structural brain abnormalities in association with cognitive deficits in schizophrenia. Schizophr. Res..

[59] Zheng F (2019). Study on the sub-regions volume of hippocampus and amygdala in schizophrenia. Quant. Imaging Med. Surg..

[60] Dieleman S, Röder CH (2013). Emotional memory modulation in schizophrenia: an overview. Acta Psychiatr. Scand..

[61] Kim H (2015). Encoding and retrieval along the long axis of the hippocampus and their relationships with dorsal attention and default mode networks: the HERNET model. Hippocampus.

[62] Spaniol J (2009). Event-related fMRI studies of episodic encoding and retrieval: meta-analyses using activation likelihood estimation. Neuropsychologia.

[63] Svoboda E, McKinnon MC, Levine B (2006). The functional neuroanatomy of autobiographical memory: a meta-analysis. Neuropsychologia.

[64] Schacter DL, Wagner AD (1999). Medial temporal lobe activations in fMRI and PET studies of episodic encoding and retrieval. Hippocampus.

[65] Zeineh MM, Engel SA, Thompson PM, Bookheimer SY (2003). Dynamics of the hippocampus during encoding and retrieval of face-name pairs. Science.

[66] Hrybouski S (2019). Involvement of hippocampal subfields and anterior-posterior subregions in encoding and retrieval of item, spatial, and associative memories: longitudinal versus transverse axis. Neuroimage.

[67] Daselaar SM (2009). Posterior midline and ventral parietal activity is associated with retrieval success and encoding failure. Front. Hum. Neurosci..

[68] Huijbers W, Pennartz CM, Cabeza R, Daselaar SM (2011). The hippocampus is coupled with the default network during memory retrieval but not during memory encoding. PloS ONE.

[69] Huijbers W (2012). Explaining the encoding/retrieval flip: memory-related deactivations and activations in the posteromedial cortex. Neuropsychologia.

[70] Kim H, Daselaar SM, Cabeza R (2010). Overlapping brain activity between episodic memory encoding and retrieval: roles of the task-positive and task-negative networks. Neuroimage.

[71] Elman JA, Rosner ZA, Cohn-Sheehy BI, Cerreta AG, Shimamura AP (2013). Dynamic changes in parietal activation during encoding: implications for human learning and memory. Neuroimage.

[72] Sestieri C, Corbetta M, Romani GL, Shulman GL (2011). Episodic memory retrieval, parietal cortex, and the default mode network: functional and topographic analyses. J. Neurosci..

[73] Yang J, Weng X, Zang Y, Xu M, Xu X (2010). Sustained activity within the default mode network during an implicit memory task. Cortex.

[74] Creem SH, Proffitt DR (2001). Defining the cortical visual systems:“what”,“where”, and “how”. Acta Psychol..

[75] Goodale MA (2011). Transforming vision into action. Vis. Res..

[76] Mishkin M, Ungerleider LG, Macko KA (1983). Object vision and spatial vision: two cortical pathways. Trends Neurosci..

[77] Kahn I, Andrews-Hanna JR, Vincent JL, Snyder AZ, Buckner RL (2008). Distinct cortical anatomy linked to subregions of the medial temporal lobe revealed by intrinsic functional connectivity. J. Neurophysiol..

[78] Behrendt R-P (2013). Conscious experience and episodic memory: hippocampus at the crossroads. Front. Psychol..

[79] Kravitz DJ, Saleem KS, Baker CI, Mishkin M (2011). A new neural framework for visuospatial processing. Nat. Rev. Neurosci..

[80] Aminoff EM, Kveraga K, Bar M (2013). The role of the parahippocampal cortex in cognition. Trends Cogn. Sci..

[81] Yonelinas AP, Ritchey M (2015). The slow forgetting of emotional episodic memories: an emotional binding account. Trends Cogn. Sci..

[82] Harvey P-O (2011). Altered dynamic coupling of lateral occipital complex during visual perception in schizophrenia. Neuroimage.

[83] Sehatpour P (2010). Impaired visual object processing across an occipital-frontal-hippocampal brain network in schizophrenia: an integrated neuroimaging study. Arch. Gen. Psychiatry.

[84] Nielsen R (2015). Second‐generation antipsychotic effect on cognition in patients with schizophrenia—a meta‐analysis of randomized clinical trials. Acta Psychiatr. Scand..

[85] Dietsche B, Kircher T, Falkenberg I (2017). Structural brain changes in schizophrenia at different stages of the illness: a selective review of longitudinal magnetic resonance imaging studies. Aust. N. Z. J. Psychiatry.

[86] Hutcheson NL (2015). Effective connectivity during episodic memory retrieval in schizophrenia participants before and after antipsychotic medication. Hum. Brain Mapp..

[87] Gurler D (2020). Neural signatures of memory encoding in schizophrenia are modulated by antipsychotic treatment. Neuropsychobiology.

[88] Association, A. P. *Diagnostic Criteria from dsM-iV-tr*. (American Psychiatric Publication, 2000).

[89] Kay SR, Opler LA, Lindenmayer J-P (1989). The positive and negative syndrome scale (PANSS): rationale and standardisation. Br. J. Psychiatry.

[90] Wechsler, D. *WASI-II: Wechsler Abbreviated Scale of Intelligence*, (PsychCorp, 2011).

[91] Potvin S, Lungu O, Tikàsz A, Mendrek A (2017). Abnormal effective fronto-limbic connectivity during emotion processing in schizophrenia. Prog. Neuropsychopharmacol. Biol. Psychiatry.

[92] Woods SW (2003). Chlorpromazine equivalent doses for the newer atypical antipsychotics. J. Clin. Psychiatry.

[93] Lang PJ, Bradley MM, Cuthbert BN (1997). International Affective Picture System (IAPS): technical manual and affective ratings. NIMH Cent. Study Emot. Atten..

[94] Power JD (2014). Methods to detect, characterize, and remove motion artifact in resting state fMRI. Neuroimage.

[95] Whitfield-Gabrieli S, Nieto-Castanon A (2012). Conn: a functional connectivity toolbox for correlated and anticorrelated brain networks. Brain Connect..

[96] Behzadi Y, Restom K, Liau J, Liu TT (2007). A component based noise correction method (CompCor) for BOLD and perfusion based fMRI. Neuroimage.

[97] Chai XJ, Castañón AN, Öngür D, Whitfield-Gabrieli S (2012). Anticorrelations in resting state networks without global signal regression. Neuroimage.

[98] Eickhoff SB (2005). A new SPM toolbox for combining probabilistic cytoarchitectonic maps and functional imaging data. Neuroimage.

[99] Amunts K (2005). Cytoarchitectonic mapping of the human amygdala, hippocampal region and entorhinal cortex: intersubject variability and probability maps. Anat. Embryol..

[100] Xia M, Wang J, He Y (2013). BrainNet Viewer: a network visualization tool for human brain connectomics. PloS ONE.

[101] Wickham, H. ggplot2: elegant graphics for data analysis (Springer-Verlag, New York, 2009, 2016).

[102] Gaser C, Dahnke R (2016). CAT-a computational anatomy toolbox for the analysis of structural MRI data. HBM.

[103] Smith SM, Nichols TE (2009). Threshold-free cluster enhancement: addressing problems of smoothing, threshold dependence and localisation in cluster inference. Neuroimage.

