# Supplementary material for: Functional connectivity abnormalities of the long-axis hippocampal subregions in schizophrenia during episodic memory
Source: NPJ Schizophr. 2021 Mar 3;7:19. doi: 10.1038/s41537-021-00147-2 (PMC7930183; doi:10.1038/s41537-021-00147-2)
Supplement: Supplementary file 1 — Supplementary Material [file 41537_2021_147_MOESM1_ESM.pdf]

- SUPPLEMENTARY MATERIAL –

Functional connectivity abnormalities of the long-axis hippocampal subregions in schizophrenia during episodic memory

Jules R. Dugré, MSc [1,2]; Alexandre Dumais, MD PhD [1,2,3]; Andras Tikasz, MSc [1,2]; Adriana Mendrek, PhD [1,4], Stéphane Potvin, PhD [1,2]

[1] Centre de recherche de l'Institut Universitaire en Santé Mentale de Montréal, Montreal, Canada

[2] Department of psychiatry and addictology, Faculty of medicine, University of Montreal, Montreal, Canada

[3] Institut national de psychiatrie légale Philippe-Pinel, Montreal, Canada

[4] Department of Psychology, Bishop's University, Sherbrooke, QC, Canada.

Corresponding author

Stéphane Potvin, PhD; Centre de recherche de l'Institut Universitaire en Santé Mentale de Montréal; 7331 Hochelaga, Montreal, Quebec, Canada; H1N 3V2; Tel. : 514-251-4015; Email: stephane.potvin@umontreal.ca

Supplementary Table 1. Difference between patients versus healthy controls on average ART's composite motion after scrubbing.

	M	SD	t	p-value
**Encoding**				
HC	0.072	0.023	2.86	0.005
SZ	0.092	0.039		
**Retrieval**				
HC	0.074	0.025	2.22	0.029
SZ	0.09	0.042		

Note. HC=Healthy Controls; SZ = Schizophrenia Patients

Supplementary Table 2. Brain regions demonstrating significant connectivity patterns between anterior and posterior Hippocampus in patients versus healthy controls with average ART's composite motion as covariate.

Regions	MNI Coordinates of Peak				p-FDR	Voxels
	Left/Right	x	y	z		
**Within Encoding Conditions**						
Diagnosis*Seeds (Left)						
dmPFC	R	6	48	26	0.044	83
Diagnosis* Seeds (Right)						
n.s.						
**Within Retrieval Conditions**						
Diagnosis*Seeds (Left)						
Precuneus/PCC	-	4	-48	38	<0.01	302
vmPFC	L	4	62	24	<0.01	238
SMG[A]	L	-32	-30	30	<0.01	285
Diagnosis*Seeds (Right)						
ITG/Temporal[B]	L	-58	2	-34	<0.01	308
Intralcalcarine cortex	L	-12	-84	10	<0.01	251

Note. [A] = cluster that included mainly the Supramarginal gyrus and some voxels encompassing the Inferior Parietal Lobule excluding the SMG; [B] = cluster that included mainly the inferior temporal gyrus and some voxels encompassing the fusiform gyrus, the anterior Parahippocampal gyrus and the uncus. dmPFC = Dorsomedial Prefrontal Cortex; PCC = Posterior Cingulate Cortex; vmPFC = Ventromedial Prefrontal Cortex; SMG = Supramarginal Gyrus; ITG = Inferior Temporal Gyrus; n.s. = not significant.
