# Supplementary material for: Functional connectivity abnormalities of the long-axis hippocampal subregions in schizophrenia during episodic memory
Source: NPJ Schizophr. 2021 Mar 3;7:19. doi: 10.1038/s41537-021-00147-2 (PMC7930183; doi:10.1038/s41537-021-00147-2)
Supplement: Supplementary file 2 — REPORTING SUMMARY [file 41537_2021_147_MOESM2_ESM.pdf]

nature research

Corresponding author(s): Stéphane Potvin

Last updated by author(s): Nov 20, 2020

Reporting Summary

Nature Research wishes to improve the reproducibility of the work that we publish. This form provides structure for consistency and transparency in reporting. For further information on Nature Research policies, see our Editorial Policies and the Editorial Policy Checklist.

Statistics

For all statistical analyses, confirm that the following items are present in the figure legend, table legend, main text, or Methods section.

n/a	Confirmed	
☐	☒	The exact sample size (*n*) for each experimental group/condition, given as a discrete number and unit of measurement
☐	☒	A statement on whether measurements were taken from distinct samples or whether the same sample was measured repeatedly
☐	☒	The statistical test(s) used AND whether they are one- or two-sided<br>*Only common tests should be described solely by name; describe more complex techniques in the Methods section.*
☐	☒	A description of all covariates tested
☐	☒	A description of any assumptions or corrections, such as tests of normality and adjustment for multiple comparisons
☐	☒	A full description of the statistical parameters including central tendency (e.g. means) or other basic estimates (e.g. regression coefficient) AND variation (e.g. standard deviation) or associated estimates of uncertainty (e.g. confidence intervals)
☐	☒	For null hypothesis testing, the test statistic (e.g. *F*, *t*, *r*) with confidence intervals, effect sizes, degrees of freedom and *P* value noted<br>*Give P values as exact values whenever suitable.*
☒	☐	For Bayesian analysis, information on the choice of priors and Markov chain Monte Carlo settings
☒	☐	For hierarchical and complex designs, identification of the appropriate level for tests and full reporting of outcomes
☐	☒	Estimates of effect sizes (e.g. Cohen's *d*, Pearson's *r*), indicating how they were calculated

Our web collection on statistics for biologists contains articles on many of the points above.

Software and code

Policy information about availability of computer code

Data collection	No Software was used
Data analysis	CONN Toolbox version 18b; CAT12; IBM SPSS Statistics 25 for Windows

For manuscripts utilizing custom algorithms or software that are central to the research but not yet described in published literature, software must be made available to editors and reviewers. We strongly encourage code deposition in a community repository (e.g. GitHub). See the Nature Research guidelines for submitting code & software for further information.

Data

Policy information about availability of data

All manuscripts must include a data availability statement. This statement should provide the following information, where applicable:

- Accession codes, unique identifiers, or web links for publicly available datasets
- A list of figures that have associated raw data
- A description of any restrictions on data availability

The data are not publicly available as they contain information that could compromise research participant privacy/consent.

Field-specific reporting

Please select the one below that is the best fit for your research. If you are not sure, read the appropriate sections before making your selection.

☐ Life sciences ☒ Behavioural & social sciences ☐ Ecological, evolutionary & environmental sciences

For a reference copy of the document with all sections, see nature.com/documents/nr-reporting-summary-flat.pdf

Behavioural & social sciences study design

All studies must disclose on these points even when the disclosure is negative.

Study description	Quantitative
Research sample	The sample of patients was recruited from the outpatient clinics from a large psychiatric hospital in Montreal in Canada (e.g. the Institut Universitaire en Santé Mentale de Montréal), and not from the community, we have no way of verifying if the current sample of schizophrenia patients is fully representative of the whole population of patients suffering from this disorder in Montreal. Demographic information about patients and controls is described in Table 1 of the manuscript.
Sampling strategy	The sample size calculation was based on previous work from one of the co-authors of the paper on emotion processing in schizophrenia (A.M.), who found large differences in brain activity between groups (d=0.8-1.0). A sample size of 40 participants per group is sufficient to detect differences of large magnitude, using a statistical threshold of 0.005, while achieving statistical power higher than 80%.
Data collection	As this is a neuroimaging study, the members of the research team who conducted the psychiatric interviews were blind to the study hypotheses. Ratings of the severity of symptoms were recorded by pen and paper.
Timing	Between september 2007 and march 2012.
Data exclusions	No data were excluded.
Non-participation	Not relevant in the current context, as this is not a randomized controlled trial.
Randomization	Not relevant in the current context, as this is not a randomized controlled trial.

Reporting for specific materials, systems and methods

We require information from authors about some types of materials, experimental systems and methods used in many studies. Here, indicate whether each material, system or method listed is relevant to your study. If you are not sure if a list item applies to your research, read the appropriate section before selecting a response.

Materials & experimental systems

n/a	Involved in the study	
☒	☐	Antibodies
☒	☐	Eukaryotic cell lines
☒	☐	Palaeontology and archaeology
☒	☐	Animals and other organisms
☐	☒	Human research participants
☒	☐	Clinical data
☒	☐	Dual use research of concern

Methods

n/a	Involved in the study	
☒	☐	ChIP-seq
☒	☐	Flow cytometry
☐	☒	MRI-based neuroimaging

Human research participants

Policy information about studies involving human research participants

Population characteristics	See Above
Recruitment	See Above
Ethics oversight	Centre de recherche de l'Institut en Santé Mentale de Montréal and the Regroupement de Neuroimagerie du Québec

Note that full information on the approval of the study protocol must also be provided in the manuscript.

Magnetic resonance imaging

Experimental design

Design type	Block Design
Design specifications	The retrieval portion consisted of viewing 48.5 s blocks of emotionally positive, negative, and neutral pictures similar to the incidental encoding task. During this second run, however, 50% of the stimuli in each block originated from the encoding task (previously viewed), while the 50% were novel (never viewed before). The order of presentation of stimuli was randomized. There were 16 s periods of rest separating the blocks from one another. Each block contained 10 images and each block type was repeated 4 times. Each picture appeared for 3000 ms followed by a blank screen with a fixation point for an average of 1.75 s (ranging from 1 to 2.5 s and giving an average inter-stimulus interval (ISI) of 4.75 s).
Behavioral performance measures	During this recognition memory task, participants were to determine, by pressing the correct button, which of the stimuli were old and which were new. (Percentage of correct response)

Acquisition

Imaging type(s)	Functional and Structural
Field strength	3T
Sequence & imaging parameters	Blood oxygen level dependent (BOLD) data was acquired using a T2-weighted gradient echo-planar imaging (EPI) sequence [repetition time (TR)=3000 ms, echo time (TE)=30 ms, flip angle=90°, matrix 64×64; voxel size=3.5mm3; 41 axial slices] on a 3.0 Tesla TRIO-TIM MRI system. The functional slices were angled parallel to the AC-PC line. An inline retrospective motion correction algorithm was employed while the EPI images were acquired. Individual high-resolution co-planar anatomical images were also acquired using a three-dimensional, spoiled gradient echo sequence (TR=19 ms; TE=4.92 ms; FA=25o; matrix size: 256x256; voxel size; 1mm3; 176 sagittal slices).
Area of acquisition	Whole

Diffusion MRI ☐ Used ☒ Not used

Preprocessing

Preprocessing software	CONN Toolbox version 18b
Normalization	Non-Linear to MNI space
Normalization template	ICBM152
Noise and artifact removal	For the preprocessing, the anatomical component-based noise correction method (aCompCor strategy), was employed to remove confounding effects from the BOLD timeseries, such as the physiological noise originating from the white matter and cerebrospinal fluid.
Volume censoring	-

Statistical modeling & inference

Model type and settings	GLM
Effect(s) tested	Diagnosis [HC versus SZ]*Condition [Retrieval versus Encoding]*Seeds [Hemisphere specific anterior versus posterior hippocampus] interaction]

Specify type of analysis: ☐ Whole brain ☒ ROI-based ☐ Both

Anatomical location(s): Probabilistic Atlas (Anatomy Toolbox)

Statistic type for inference (See Eklund et al. 2016)	voxel-wise p<0.005
Correction	FDR correction p<0.05

Models & analysis

n/a	Involved in the study
☐	☒ Functional and/or effective connectivity
☒	☐ Graph analysis
☒	☐ Multivariate modeling or predictive analysis

Functional and/or effective connectivity	Bivariate Regression (weighted least squares linear model)
